# Bibliometric and visual analysis of miRNAs in heart diseases from 2004 to 2023

**DOI:** 10.3389/fcvm.2025.1465646

**Published:** 2025-03-20

**Authors:** Ying Jin, Jingqi Duan, Qiaoxiang Yin, Yanmin Ma, Jingli Lou, Wei Zhang

**Affiliations:** Department of Geriatrics, Air Force Medical Center, PLA, Beijing, China

**Keywords:** miRNA, heart disease (HD), VOSviewer, CiteSpace, visual analysis

## Abstract

**Background:**

MicroRNAs (miRNAs) add a new dimension to HD forecast, diagnosis, and therapy based on the potential applications. The miRNA-related research in the heart disease (HD) field has received close attention in the past two decades. However, there is a lack of studies that comprehensively and objectively analyze the current situation of miRNA application in the HD field using the bibliometrics method.

**Objective:**

To comprehensively analyze the global scientific outputs of miRNAs in HD research from 2004 to 2023.

**Methods:**

All the articles and reviews of miRNA-related research in the HD field were retrieved using the Web of Science core collection (WOSCC) title search, and bibliometric analysis was performed in Microsoft Excel 2019, CiteSpace, VOSviewer, and Bibliometrics (R-Tool of R-Studio).

**Results:**

3,874 publications were included in the bibliometric analysis. Collaborative network analysis indicates that China with the maximum number of publications (2,063) and the USA with the highest total citations (59,331) are influential countries in this field. Peking Union Medical College is the most prolific university with the maximum publications (134), and the University of California System is the most authoritative institution regarding betweenness centrality (0.27). PLOS ONE tops the journal list of publications, closely followed by the International Journal of Molecular Sciences and Scientific Reports with more than 100 articles. Considering the number of publications, citations, and total link strength overall, Olson. Eric N, Van Rooij Eva, Thum Thomas, Yang Baofeng, Wang Kun; and Lu Yanjie are authoritative authors in this field. The expression changes and regulatory mechanisms of specific miRNAs in various heart biological and pathophysiological processes have been the continuous research hotspots. “exosomes”, “extracellular vesicles”, “autophagy”, and “management” have been novel hot research topics since 2018, which focused on the diagnosis and treatment of HD. The current research development trend is how to translate the achievement of miRNA-related diagnosis and therapeutic drugs for HD into the clinic.

**Conclusion:**

Our study revealed the intellectual structure of miRNA in HD research, which may help scholars understand this field comprehensively and find partners.

## 1 Introduction

Heart diseases (HD) usually present as disorders of the coronary artery, myocardium, electrocardiogram pathway, etc. Cardiovascular disease (CVD) is still the leading cause of death throughout the world. However, approximately one-quarter reduction in mortality is expected by 2025, mostly related to the appearance of novel diagnostic techniques and therapies (1).

MicroRNAs (miRNAs) are small noncoding RNAs (∼23 nucleotides) that regulate gene expression by pairing to mRNAs at the post-transcriptional level in plants and animals (2). Functionally, miRNAs provide transcriptional control and play an important role in normal physiological development and pathological conditions.

Abundant studies on miRNAs in the development and progression of heart conditions implicate hope in disease diagnosis, prognosis, and treatment. Humans after birth have a limited capacity to regenerate heart tissue after injury (3). miRNA-related research brings the therapeutic potential for human heart injury by various diseases. The study discusses the current status and trends of miRNA-related research in various cardiac conditions, including ischemic HD, cardiac hypertrophy, arrhythmia, etc.

Bibliometric analysis involves analyzing published works objectively on an academic area over a specific period using mathematical and statistical methods, both qualitatively and quantitatively (4, 5). This method provides an objective view of the status, hotspots, trends, and frontiers by analyzing countries, institutions, journals, authors, and keywords of published works related to the specific research field (4, 6).

Despite the rapid universality of miRNA-related research in the last twenty years, there is still a lack of bibliometric analyses of “miRNAs in HD” research. Therefore, this study analyzed the overall situation of “miRNAs in HD” research in the past two decades with VOSviewer (7), CiteSpace (8) software programs, and R software. This may help scholars gain insight into the corresponding academic fields and find collaborations.

## 2 Materials and methods

### 2.1 Data sources

The raw data of this study were exported from the Web of Science Core Collection (WOSCC) database, a comprehensive, fundamental data source widely used for bibliometric analysis and information mapping in academia covering a large part of the medical literature (9).

### 2.2 Data collection

**Figure 1:**
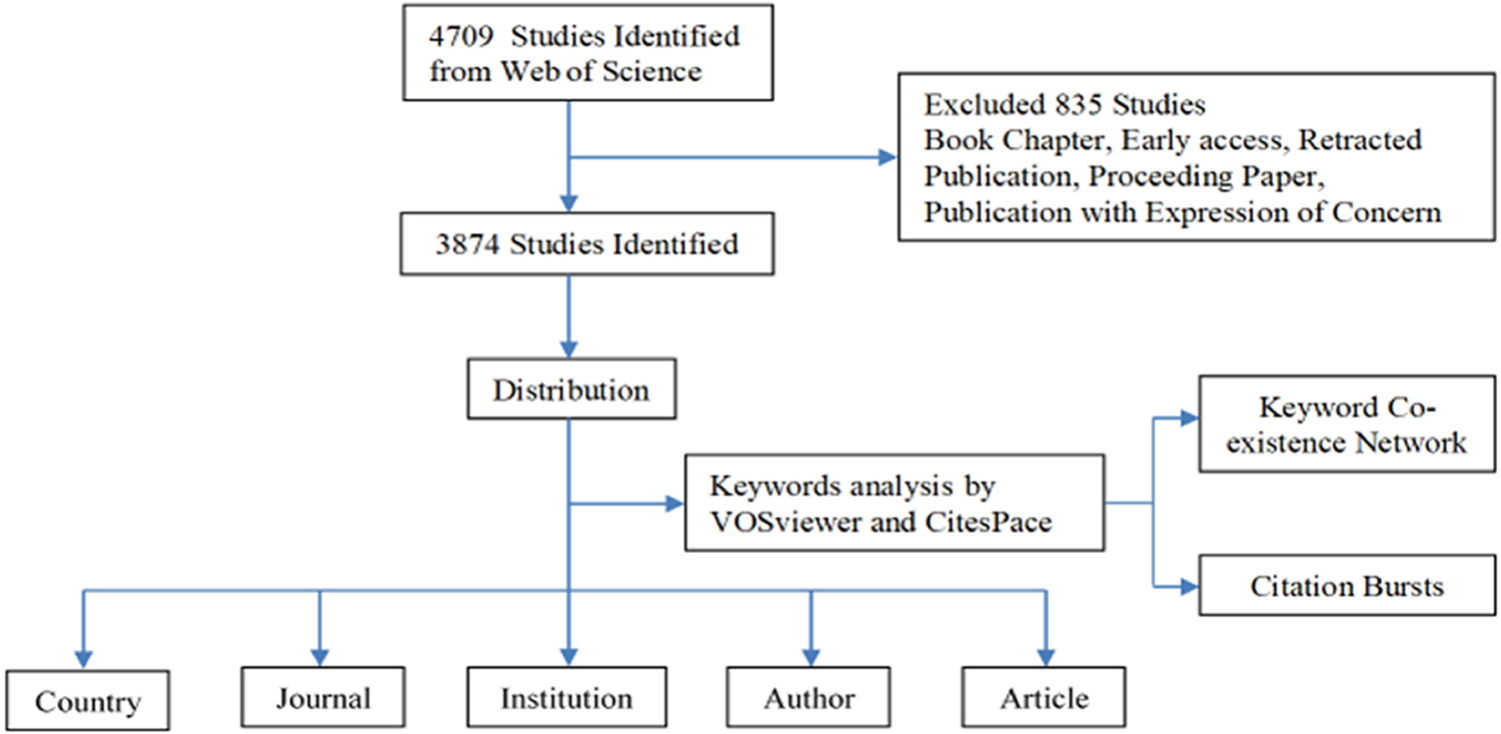
The inclusion and exclusion of the "miRNAs in HD" research.

In the WOSCC database, TS and TI stand for Topic and Title, respectively. For precise literature retrieval, we utilized title retrieval. The search format presented below: TI = (“miRNA*” OR “microRNA*” OR “miR*” OR “micro ribonucleic acid*” OR “micro RNA*” OR “RNA micro”) AND TI = (“heart” OR “cardi*” OR “coronary” OR “atrium*” OR “atrial” OR “ventricle*” OR “arrhythmia*”) AND PY = (2004–2023) AND LA = (English). The literature publication time was limited to December 31, 2023. The language was limited to English, “Article” was selected as the article or review type, and the retracted publication was excluded, resulting in 3,874 articles (Figure 1). The data was downloaded as plain text files in text formats, according to the above retrieval strategy. The search was completed on February 29, 2024. In the data-collecting process, the results were verified by two researchers separately. A third-party adjudication resolved discrepancies and was immediately followed by a three-way harmonization.

### 2.3 Bibliometrics analysis

CiteSpace and VOSviewer are currently widely used software for bibliometric analysis and bibliometric network graph analysis (10), respectively. We used CiteSpace 6.2.R4 advanced visualization and VOSviewer 1.6.20 to visually analyze the overall network of journals, institutions, authors, references, and key nodes via the data files downloaded from the WOSCC database.

In addition, we used Bibliometrix (R-Tool of R-Studio) (11) to visually analyze the distribution of countries/regions, references, and keywords, and Microsoft Excel 2019 to show the publication and citation situation of the literature over the 20 years. All data in this study were obtained from public databases; therefore, ethical review was unnecessary.

Using VOSviewer, clustering analyses of nodes representing various countries/regions, journals, authors, and keywords were performed based on their occurrence in the download data. The circle's color, size, and connecting lines were used to describe the frequency and links of nodes. Moreover, analyses on countries/regions, journals, and keyword citation bursts were also performed by CiteSpace. The betweenness centrality refers to the core position of the nodes. The strength indicates the connection tightness between object data. The time distribution of keywords is described by beginning and end times.

## 3 Results

### 3.1 General information

**Figure 2:**
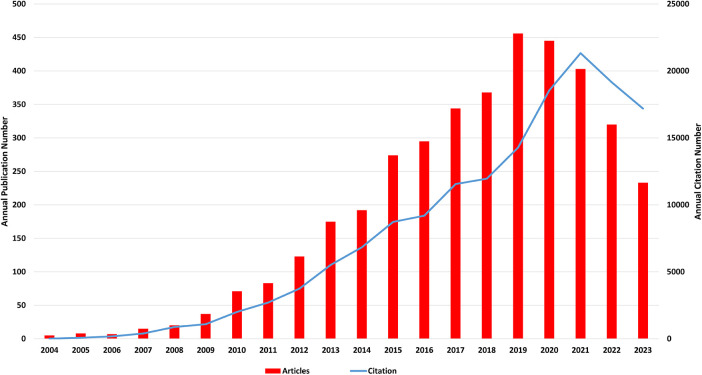
Annual number of publications and citations of "miRNAs in HD" research.

There were 3,874 documents (3,381 articles and 493 reviews) on “miRNA in HD” in the WOSCC database from 2004 to 2023 (Figures 1, 2). The distribution of the top ten research areas according to WOS, the top 10 academic categories is reported in Table 1. It shows that 905 publications were included in the subject area “Cardiovascular Systems Cardiology” (23.361%), 778 in “Cell Biology” (20.083%), 704 in “Medicine Research Experimental” (18.172%), 580 in “Biochemistry Molecular Biology” (14.972%), and 388 in “Pharmacology Pharmacy” (10.015%), etc. The mean citation frequency per article is 40.36, with an H-index of 161. In-depth analysis indicates that the top 50 articles, in terms of citation frequency, accounted for 19.8% of the total citations, with an average of 613.5 citations per article.

**Table 1:** Research areas of “miRNAs in HD”.

Research areas	Articles	% of 3,874
Cardiovascular System Cardiology	905	23.361
Cell Biology	778	20.083
Research Experimental Medicine	704	18.172
Biochemistry Molecular Biology	580	14.972
Pharmacology Pharmacy	388	10.015
Science Technology Other Topics	312	8.054
Oncology	220	5.679
Physiology	210	5.421
Genetics Heredity	194	5.008
General Internal Medicine	158	4.078

### 3.2 Statistical analysis of annual global literature publication and citation

Based on the data gathered from the WOSCC database, from 2004 January 1 to 2023 December 30, the enrolled 3,874 publications have received a cumulative total of 156,334 citations. The annual number of publications and citations for “miRNAs in HD” research is exhibited in Figure 2. Generally, the yearly volume of articles showed a rapid upward trend with a peak in 2019 (456) and a significant downward trend from then on. Similarly, the number of annual citations shows the same curve, reaching the inflection point of 21,329 in 2021.

### 3.3 Quantity and citations of different countries/regions

Table 2 shows the top 20 countries/regions by publication quantity. Meanwhile, it shows the corresponding centrality, frequency of citations, and year when the first document was published. In miRNA-related studies on HD, China has the highest number of publications (2,063) and the highest number of total citations (61,164), followed by the USA (751, 59,331) and Germany (233, 15,957). The Netherlands has the highest average citation of all the countries/regions (84.6 times), followed by the USA (79 times) and Germany (68.5 times). The betweenness centrality of countries/regions is a critical indicator to identify the role of the countries/regions in the global network. Based on this indicator the USA has the highest centrality (0.46), England (0.24), and Germany (0.17) ensued. The USA has established a leading role regarding average citation and betweenness centrality in this field.

**Table 2:** Top 20 countries/regions in terms of publications of “miRNAs in HD” research.

Rank	Countries/regions	Publications	Centrality	Citations	Year	Average citation
1	China	2,063	0.15	61,164	2007	29.6
2	USA	751	0.46	59,331	2004	79.0
3	Germany	233	0.17	15,957	2004	68.5
4	Italy	191	0.11	12,586	2007	65.9
5	England	127	0.24	8112	2010	63.9
6	Netherlands	118	0.06	9,979	2007	84.6
7	Spain	97	0.08	2595	2011	26.8
8	Japan	96	0.02	4,877	2009	50.8
9	Canada	94	0.03	5,928	2007	63.1
10	Iran	80	0.03	1,347	2014	16.8
11	Australia	76	0.09	4,002	2006	52.7
12	Poland	67	0.01	1,758	2013	26.2
13	Brazil	63	0.01	1,651	2005	26.2
14	Taiwan	54	0.05	1,407	2011	26.1
15	South Korea	53	0	1,331	2010	25.1
16	Sweden	53	0.03	2,712	2004	51.1
17	India	49	0.07	940	2011	19.2
18	France	45	0.04	2,307	2006	51.3
19	Denmark	35	0.02	1,482	2010	42.3
20	Czech Republic	34	0.01	1,407	2014	41.4

### 3.4 Distributions by countries/regions

**Figure 3:**
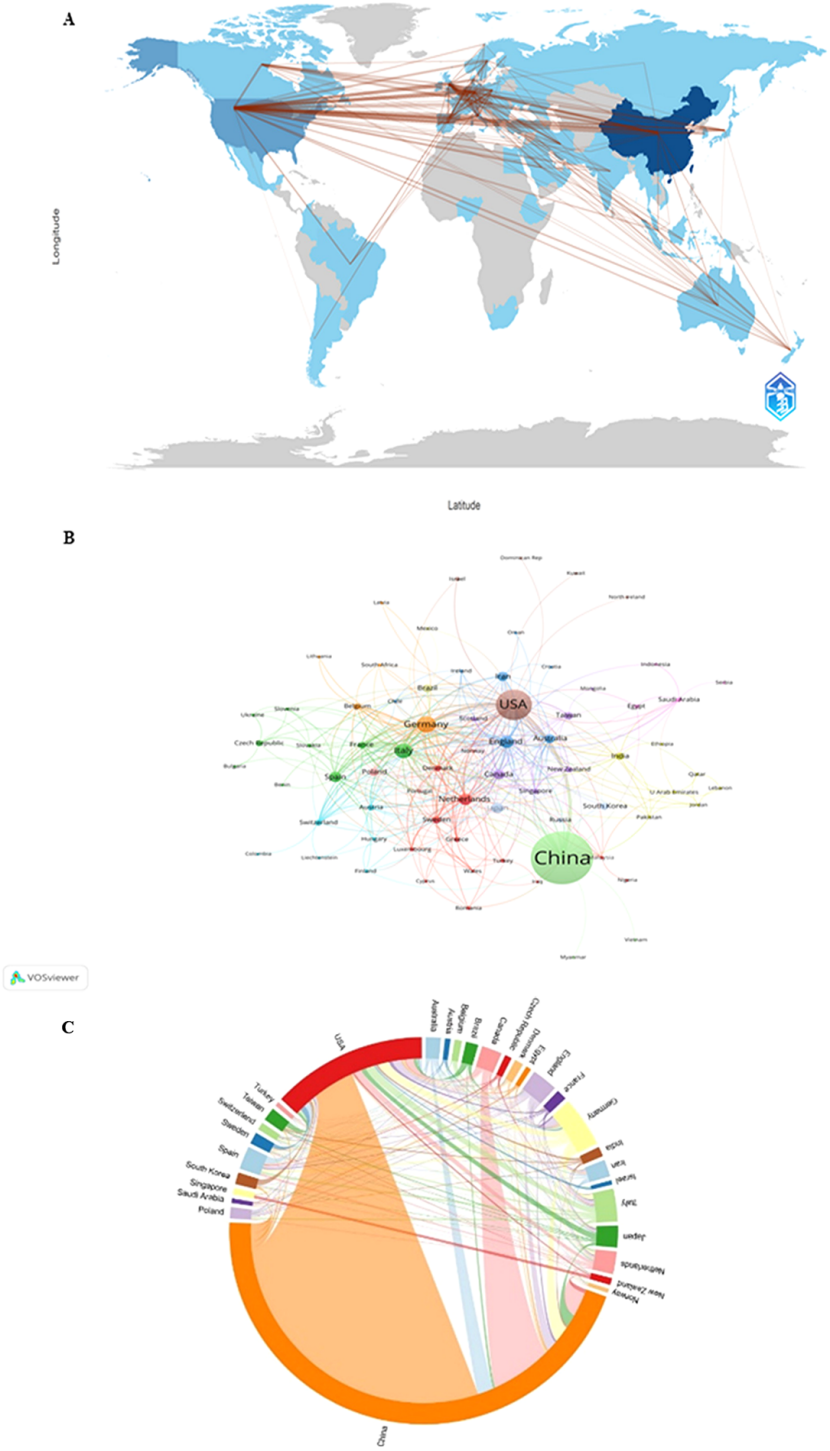
Analysis of country/region network in the "miRNAs in HD" research. **(A)** Countries/regions collaboration map in R software. **(B)** Collaborative network visualization of countries/regions in VOSviewer. The figure shows the countries/regions distribution of miRNA-related literature on heart disease. The size of the nodes indicates their literature quantity. The number of connecting lines is positively proportional to the closeness between different countries/regions. **(C)** Collaborative network visualization of countries/regions in VOSviewer.

In review China and the USA were the most prominent countries, with the highest publications and citations. Currently, 76 countries/regions are involved in miRNA-related research of heart conditions, mainly located in East Asia, Oceania, North America, and Europe, with strong links among them (Figure 3A). As indicated in Figure 3A, the numbers of connecting lines indicate the mutual links between different countries/regions. Among lots of countries/regions, there is deep cooperation (Figure 3B). The closest relationship in terms of country-to-country cooperation is between China and the USA (Figure 3C). In addition, the USA also collaborates closely with Italy, the United Kingdom, Canada, Japan, Germany, Netherlands, Japan, Brazil, Australia, India, Sweden, Poland, Spain, and France, etc. It is the same situation between China and other countries/regions including Canada, the Netherlands, Japan, Singapore, Australia, and the United Kingdom. The visualization in VOSviewer of the countries/regions with miRNA-related literature of HD shows their cooperation similarly (Figure 3B). The network collaboration analysis shows that countries/regions are divided into 12 clusters in VOSviewer according to the cooperation closeness, marked with different colors. In Figure 3B, each node represents a country/region, and the node's radius expands with the increase of its document volume.

### 3.5 Distribution by institutions

Table 3 shows the top 20 institutions in terms of number of publications. Together their frequency of citations and the corresponding centrality are listed in the table. The institution with the highest number of publications is Peking Union Medical College (134), followed by Harbin Medical University (131) and Nanjing Medical University (100). This table shows fifteen institutions in China, followed by the USA with three and Germany with two institutions. Among the top 20 institutions, the University of California System (0.27), Harvard University (0.21), Harvard Medical School (0.1), and Hannover Medical School (0.1) show high centrality, which implies that these institutions have a significant effect on the development of the “miRNAs in HD” research.

**Table 3:** Top 20 institutions in terms of number of publications.

Rank	Institution	Publications	Centrality	Year
1	Peking Union Medical College	134	0.03	2009
2	Harbin Medical University	131	0.03	2007
3	Nanjing Medical University	100	0.03	2011
4	Shanghai Jiao Tong University	94	0.04	2010
5	Harvard University	84	0.21	2004
6	University of California System	83	0.27	2004
7	Capital Medical University	77	0.06	2014
8	Fudan University	74	0.03	2011
9	Huazhong University of Science & Technology	74	0.03	2010
10	Central South University	69	0.04	2013
11	Harvard Medical School	61	0.1	2004
12	Hannover Medical School	56	0.1	2007
13	Jilin University	55	0	2015
14	Tongji University	51	0.03	2012
15	Zhengzhou University	50	0	2015
16	Fu Wai Hospital - CAMS	49	0	2009
17	Qingdao University	49	0.01	2015
18	Southern Medical University - China	48	0.02	2010
19	German Centre for Cardiovascular Research	47	0.09	2013
20	Chinese Academy of Sciences	46	0.03	2009

**Figure 4:**
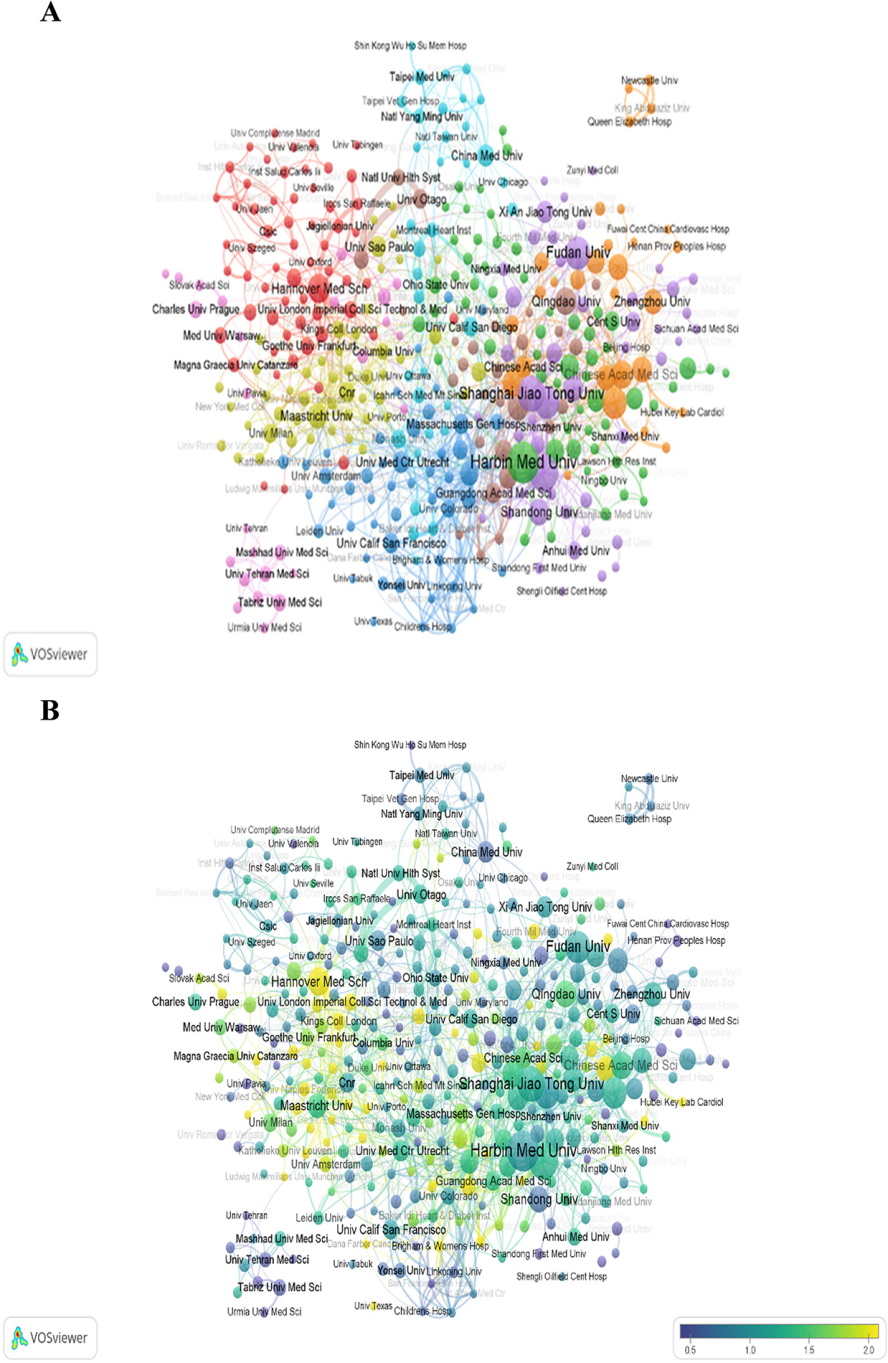
Analysis of institutions of the "miRNAs in HD" research in VOSviewer. **(A)** Collaborative network visualization of institutions with more than 25 documents. **(B)** Overlay visualization of institutions in VOSviewer. The number of articles published by institutions in 5 years from 2016 to 2020. The number of publications of the institution in the 5 years divided by the total publications from 2004 to 2003 is its heat value of the 5 years.

The institution analysis aims to explore the global distribution of miRNA-related research of HD and help scholars seek cooperation. Using VOSviewer to plot institution collaboration network graphs, institutions with at least 25 documents cooperation are divided into 9 closely related blocks (Figure 4A). Harbin Medical University, Nanjing Medical University, and Shanghai Jiao Tong University are relatively highly productive institutions in the institutional cooperation network (Figure 3A), but their centralities are relatively low (Table 3).

Figure 4B reveals the ratio of institutional publications to total publications from 2016 to 2020, a time when the studies of this field rapidly burst. The ratio numbers were achieved by dividing the publications amount of the five years in this field of each institution by their total amount of publications from 2004 to 2023. The closer the node color is to yellow, the higher the ratio. The high ratio indicates that the corresponding institution is an emerging force in this field in the five years. The figure shows that Hannover Medical School, the University of Texas, Fourth Military Medicine University, and some other institutions with yellow nodes have achieved rapid growth, having issued lots of papers much more than residual institutions during the five years. In contrast, the documents issued by Yonsei University, Shandong University, Tabriz University of Medical Sciences, China Medical University, Harbin Medical University, and University Texas Health Science Center of Houston represented by purple nodes are relatively few.

### 3.6 Distribution by journals

A total of 78 journals were involved in the “miRNAs in HD” research. The bibliometric online analysis platform identified journals with high publication volume and impact on the “miRNAs in HD” research.

The top 20 journals by publication volume with a total of 1,195 documents (30.85%) are listed in Table 4. Meanwhile, the table shows the corresponding total citations, average citations, the IF, the H-index, and the JCR category quartile of the above 20 journals in this field from 2004 to 2023. PLOS ONE (134, Q2, H-index 44) with the highest number of publications and H-index ranks first in this table. Two journals, International Journal of Molecular Sciences (119, Q2, H-index 16) and Scientific Reports (100, Q1, H-index 32) rank second and third in this table, respectively.

**Table 4:** Top 20 journals in terms of the number of publications of “miRNAs in HD”.

Rank	Journal	Publications	Total citations	Average citations	JCR quartile	IF	H-index
1	PLoS One	134	5,089	37.98	Q2	3.7	44
2	International Journal of Molecular Sciences	119	1,945	16.34	Q1	5.6	16
3	Scientific Reports	100	2,998	29.98	Q2	4.6	32
4	Molecular Medicine Reports	75	1,518	20.24	Q3	3.4	24
5	Journal of Molecular and Cellular Cardiology	70	4,810	68.71	Q2	5	40
6	Frontiers in Cardiovascular Medicine	66	599	9.08	Q2	3.6	15
7	European Review for Medical and Pharmacological Sciences	61	1,111	18.21	Q2	3.3	18
8	Experimental and Therapeutic Medicine	61	717	11.75	Q3	2.7	16
9	Circulation Research	59	12,405	210.25	Q1	20.1	52
10	Biochemical and Biophysical Research Communications	56	2,217	39.59	Q2	3.1	26
11	Biomed Research International	48	1,611	33.56	Q3	3.246	21
12	Cellular Physiology and Biochemistry	45	2,045	45.44	Q1	5.5	30
13	Cardiovascular Research	42	4,316	102.76	Q1	10.9	30
14	Oxidative Medicine and Cellular Longevity	42	1,001	23.83	Q2	7.31	18
15	International Journal of Cardiology	40	2,209	55.23	Q2	3.5	28
16	International Journal of Clinical and Experimental Pathology	39	449	11.51	Q4	1.4	11
17	Journal of Cellular Physiology	37	1,225	33.11	Q1	5.6	21
18	Cells	36	498	13.83	Q2	6	11
19	Journal of Cardiovascular Pharmacology	33	522	15.82	Q3	3	13
20	Journal of Cellular and Molecular Medicine	32	2,247	70.22	Q2	5.3	25

As Table 4 shows, five journals are distributed in quartile 1 (Q1), ten journals in quartile 2 (Q2), four journals in quartile 3 (Q3), and one journal in quartile 4 (Q4). Circulation Research has the highest journal IF (20.1). Cardiovascular Research comes second with IF 10.9. Other journals rank behind, including seven with IF between 5 and 10, and eleven with IF less than 5. Both Circulation Research (IF 20.1, H-index 52, Average Citations 210.25) and Cardiovascular Research (IF 10.9, H-index 30, Average Citations 102.76) are in quartile 1(Q1), with a relatively high IF value, H-index, and average citations, implying a strong impact of the two journals in this field.

**Figure 5:**
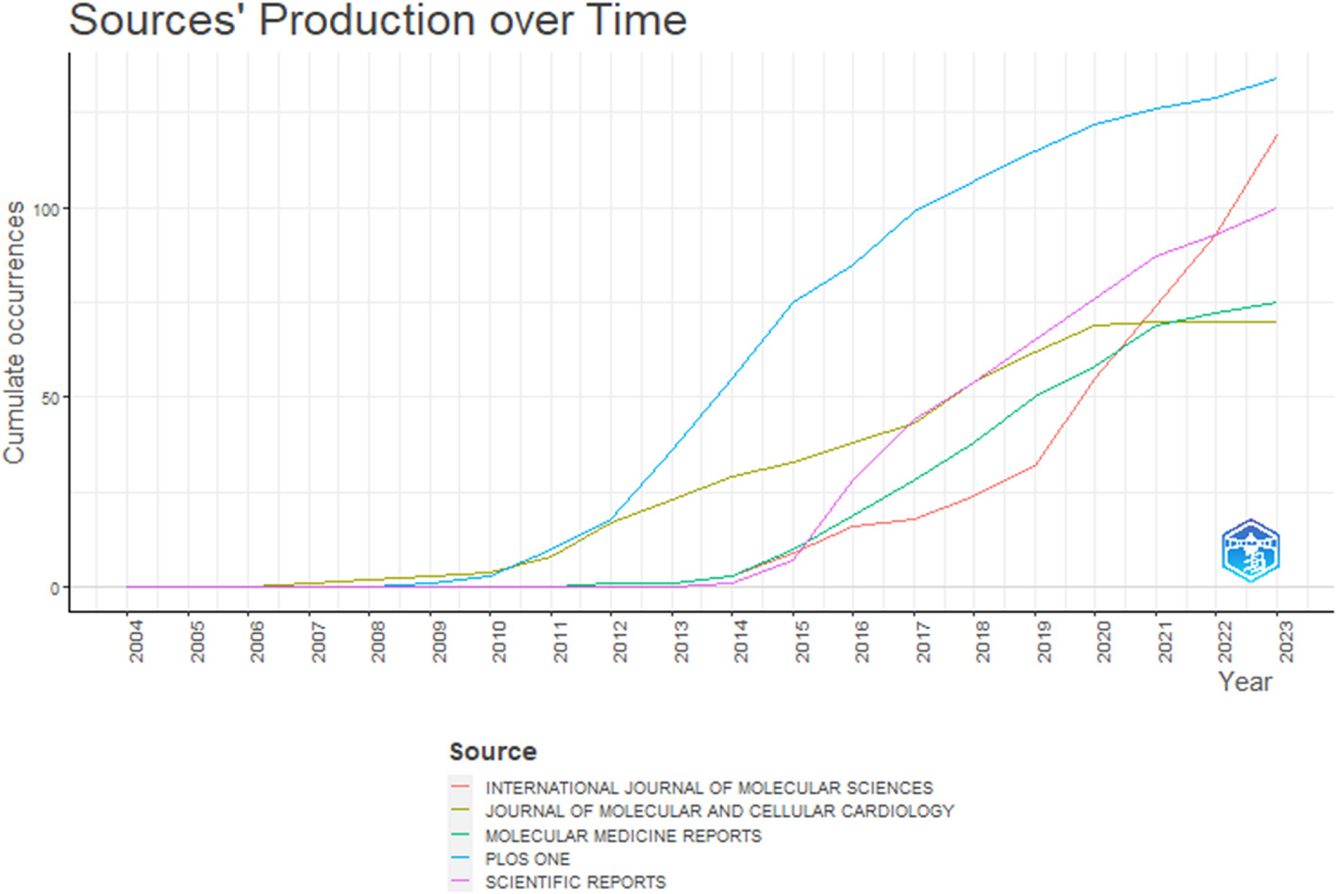
Institution production over time of the "miRNAs in HD" research in R software.

The top 5 journals in terms of the number of publications were analyzed furtherly, and we plotted the cumulative publication number-time curve (Figure 5), including International Journal of Molecular Sciences, Journal of Molecular and Cellular Cardiology, Molecular Medicine Reports, PLOS ONE, and Scientific Reports. The result shows that the Journal of Molecular and Cellular Cardiology didn't publish any article in this field in the past two years. However, the number of articles in the other four journals, with an obvious upswing trend continuously during the last decade, suggests that the four journals prefer to publish relevant articles in this field currently. A total of twenty-six articles, the most publications among all the journals in the field in 2023, were published in International Journal of Molecular Sciences, indicating that it is the most popular journal for this academic field in the last year.

**Figure 6:**
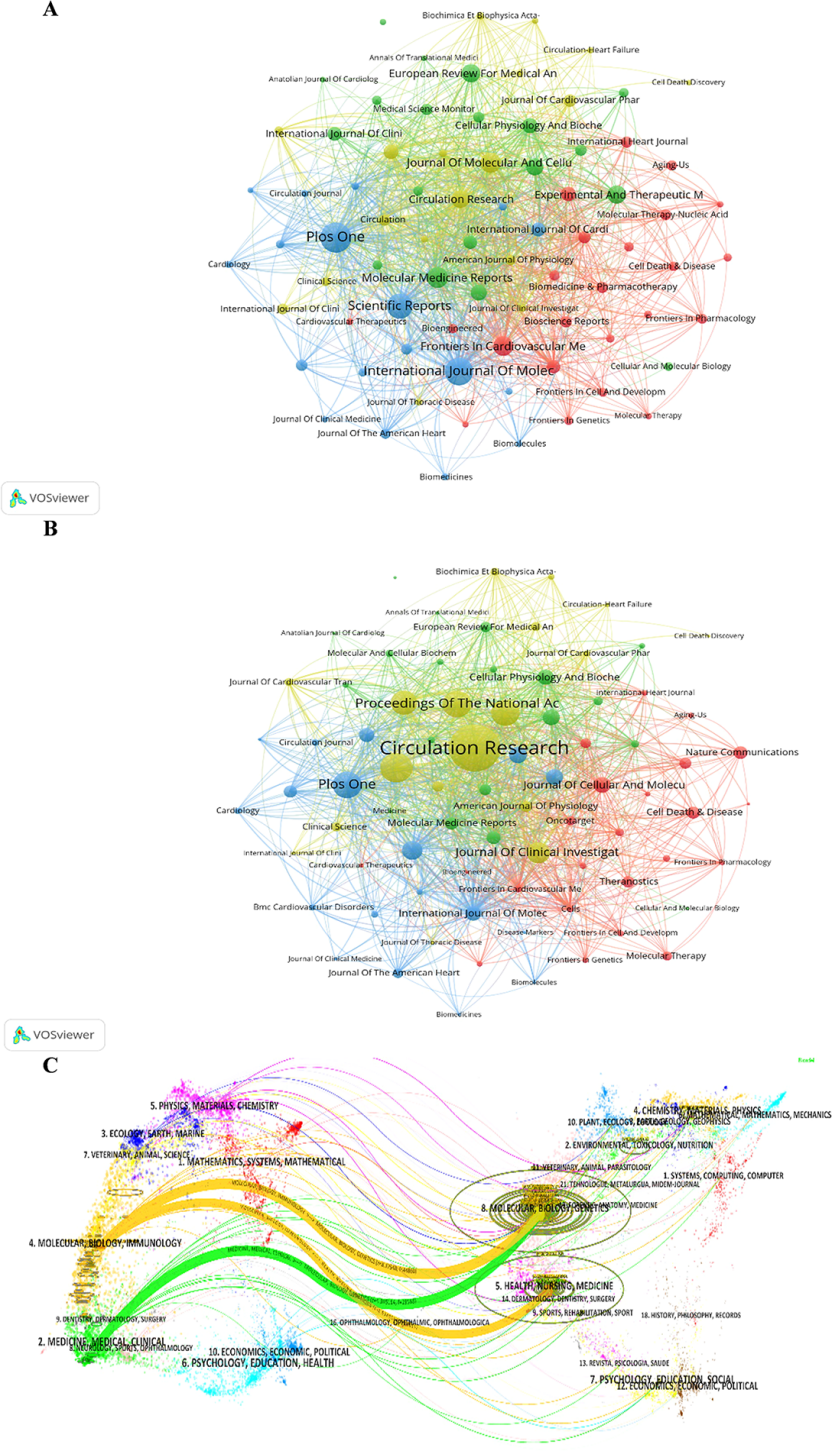
Journal analysis of the "miRNAs in HD" research. **(A)** Analysis of collaborative network visualization of journals in VOSviewer. The figure shows the journals with more than 10 documents. The colors of the nodes represent the journals in different clusters, and the size of the nodes indicates the frequency of their occurrence. **(B)** Analysis of collaborative network visualization of journals' citations in VOSviewer. **(C)** The dual-map overlay of journals on knowledge flow analysis in Citespace. Citing journals are on the left, cited journals are on the right, and colored paths indicate citation relationships.

Figures 6A,B show the collaborative networks among the journals, in which the miRNA-related articles of HD were published. The clustering is based on the similarity of the journals and divided into 4 categories with different colors. The node size in the two figures indicates the number and the total citations of related articles in the corresponding journal, respectively. The journals of the blue cluster are devoted to clinical areas. The journals of the green cluster focus on physiology and biochemistry. The journals of the red cluster are mainly pharmacology and pharmacotherapy. The journals of the yellow cluster are centered on pathology.

The evolution of knowledge citations and co-citation between citing and cited journals was explored through the knowledge trajectory graph in Figure 6C. The dual-map overlay of journals shows the research topic distribution, citation trajectory, and research area changes in academic journals. Colored arcs connecting the citing journals on the left and cited journals on the other side indicate citation trajectory. The citing journals mainly include the issues of MATHMATICS, SYSTEMMS, MATHMATICAL, MOLECULAR, BIOLOGY, IMMUNOLOGY, MEDICINE, MEDICAL, and CLINICAL. The cited journals mainly include the issues of MOLECULAR, BIOLOGY, GENETICS, HEALTH, NURSING, MEDICINE, PSYCHOLOGY, EDUCATION, and SOCIAL. The cited topics have built the foundation, and the citing issues have formed the frontiers of the “miRNAs in HD” research.

### 3.7 Author collaboration network graph

In VOSviewer, co-cited authorship refers to the literature of two authors being cited by a third author simultaneously. The co-citation frequency reflects academic focus and research density. Table 5 shows the top 20 authors according to the number of publications, together with the corresponding institutions, citation times, and total link strength. The author with the highest number of publications is Thum Thomas (Cardior Pharmaceut GmbH, Germany) (47), followed by Yang Baofeng (Southeast University, China) (28), Shan Hongli (Royal Netherlands Academy of Arts & Sciences, Netherlands) (25), Lu Yanjie (Chengde Medical University, China) (24), and Xiao Junjie (Shanghai University, China) (22). Olson, Eric N (University of Texas, USA) (10,966) is the author who is most frequently cited, followed by Van Rooij Eva (University of Texas, USA) (7,779), Thum Thomas (Cardior Pharmaceut GmbH, Germany) (6,311), Condorelli, Gianluigi (Univ Milan, Italy) (3,852), and Yang, Baofeng (Southeast University, China) (3,630). It is worth noting that Olson Eric N, Van Rooij Eva, Thum Thomas, Yang Baofeng, Shan Hongli, and Lu Yanjie have a high volume of articles, a high number of citations, and strong total link strength at the same time, indicating their important effect in the “miRNAs in HD” field.

**Table 5:** Top 20 authors in terms of numbers of documents.

Rank	Author	Documents	Citations	Total link strength	Institutions
1	Thum, Thomas	47	6,311	99	Cardior Pharmaceut GmbH (Germany)
2	Yang, Baofeng	28	3,630	165	Southeast University (China)
3	Shan, Hongli	25	2,287	161	Royal Netherlands Academy of Arts & Sciences (Netherlands)
4	Lu, Yanjie	24	3,462	151	Chengde Medical University (China)
5	Xiao, Junjie	22	1,563	105	Shanghai Univ (China)
6	Wang, Yan	21	572	46	Zunyi Med Univ (China)
7	Chen, Chen	20	910	25	Tongji Hospital, Tongji Medical College (China)
8	Olson, Eric N.	20	10,966	98	University of Texas (USA)
9	Zhang, Yong	20	1,782	119	Gladstone Institute of Cardiovascular Disease (USA)
10	Wang, Dao Wen	19	873	62	Henan Agricultural University (China)
11	Wang, Kun	19	2,726	96	Qingdao Univ (China)
12	Li, Jun	18	374	39	Anhui Med Univ (China)
13	Van Rooij, Eva	17	7,779	24	University of Texas (USA)
14	Ge, Junbo	16	695	72	Fudan Univ (China)
15	Li, Lang	16	338	36	Guangxi Med Univ (China)
16	Sluijter, Joost P. G.	16	1,440	105	Univ Med Ctr Utrecht (Netherlands)
17	Wang, Jun	16	429	12	Texas A&M Syst Hlth Sci Ctr (USA)
18	Xu, Chaoqian	16	2,162	20	Harbin Med Univ (China)
19	Zhang, Jing	16	493	25	Henan Univ (China)
20	Zhang, Yan	16	582	13	Harbin Med Univ (China)

**Figure 7:**
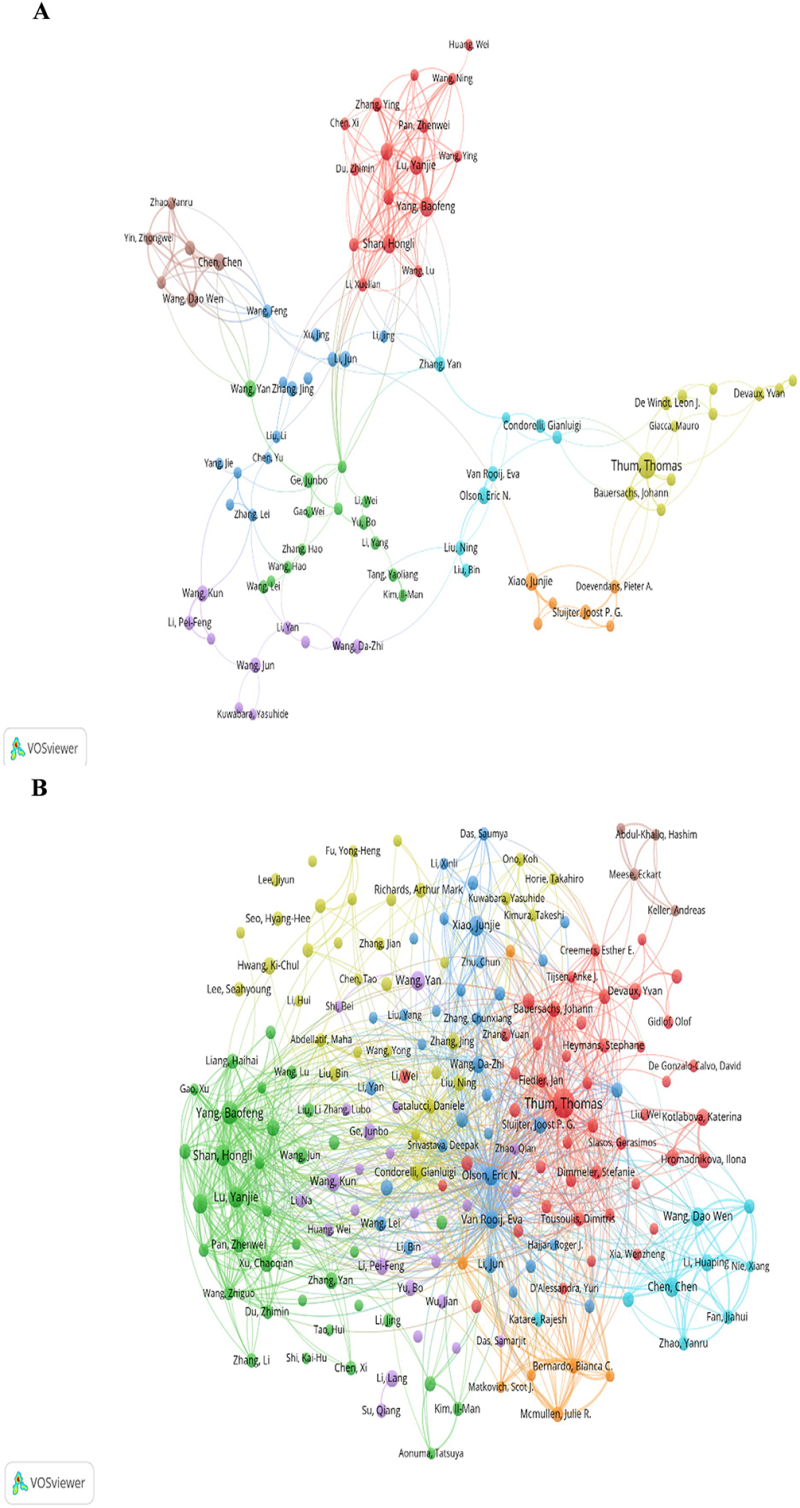
Authors analysis of the "miRNAs in HD" research in VOSviewer. **(A)** Authors collaborative network. **(B)** Authors citations collaborative network.

In VOSviewer, Figure 7A shows the collaborations of the authors in this field. A total of 110 authors with more than 10 published articles formed 8 clusters. Authors in various clusters are identified by the nodes in different colors, and their frequency of occurrence is determined by their size. Authors have deep cooperation between them inside a cluster. In addition, there are also one or two authors in each cluster connecting closely with other authors outside their clusters. Thum Thomas has a close relationship with Condorelli, Gianluigi, Doevendans, Pieter A, and, Devaux, Yvan, respectively. Yang, Baofeng has active connectivity with Zhang, Yan, Zhang, Li, and Li, Jing. While Chen Chen is in close collaboration with Zhang Yan, Wang Feng, and Wang Yan. Wang Kun is associated with Zhang, Yuan, and Zhang, Lei.

Figure 7B shows the co-cited authorship network of the “miRNA in HD” research. The authors with no less than 8 citations are mainly divided into 8 clusters: Thum, Thomas, Fiedler, Jan, Bauersachs, Johann, et al. (red); Olson, Eric N, Wang, Da-zhi, Van, Rooij E, et al. (dark blue); Wang Dao Wen, Chen Chen, Zhao Yanru, et al. (light blue); Yang, Baofeng, Lu, Yanjie, Shan, Hongli, et al. (green); Condorelli Gianluigi, Catalucci, Daniele, Liu, Ning, et al. (yellow); Ge, Junbo, Wang, Kun, Wang, Yan, et al. (purple); Bernardo, Bianca C, Mcmullen, Julie R, Matkovich, Scot J, et al. (orange); Keller, Andreas, Abdul-khaliq, Hashim, Meese, Eckart, et al. (brown).Olson Eric N, Van Rooij E, Thum Thomas, Lu, Yanjie, and Yang Baofeng are prominent nodes of the collaborative network and have plenty of contacts between them. Olson Eric N, Van Rooij E, and Thum Thomas are at the center of the whole network, with direct connections to the authors in other clusters. While Yang Baofeng and Lu, Yanjie have relatively less active contact with the authors outside their cluster.

### 3.8 Keyword analysis

A total of 9,681 keywords were obtained from the raw data, which could be divided into 5 clusters. Table 6 shows the top 20 keywords by their occurrences in the articles in the “miRNAs in HD” field, implying the popular research topics in this field. The keyword with the highest frequency is “micrornas” (2,075), followed by “expression” (1,286). Moreover, “heart failure” (759), “apoptosis” (654), “biomarker” (639), “heart” (544), and “inflammation” (478) are also frequent keywords, implying their corresponding researches are hot study topics in this field. After overviewing the corresponding articles, we found that the keywords usually connecting “miRNAs” with heart conditions, such as “heart failure”, “myocardial infarction”, “cardiovascular disease”, and “cardiac hypertrophy”, etc.) are “apoptosis”, “proliferation”, “cancer”, “inflammation”, “expression”, “biomarker”, “circulating microrna”, “gene expression”, “cell”, “mechanisms”, and “activation”, etc. Table 7 shows the top 20 keywords with the strongest citation bursts, ranked by beginning year. In the table, the “year” indicates the average time of keyword appearance. The “begin” and “end” indicate the beginning and ending time of the keyword citation burst occurrence, respectively. The keywords including “muscle specific microrna”, “*in vivo*”, and “gene expression” have a higher citation burst with a strength value above 15, indicating the keyword's high occurrence frequency during the specific period. The keywords including “autophagy” (from 2018), “diabetic cardiomyopathy” (from 2019), “cardiac dysfunction” (from 2019), “extracellular vesicles” (from 2019), “long noncoding rna” (from 2018), “exosomes” (from 2020), and “management” (from 2021), have strong citation bursts till 2023, suggesting the study novel hot frontiers in this field recently. The articles on keywords of “exosomes” and “extracellular vesicles” were focused on their carrier role of miRNAs as potential biomarkers or treatment of HD, and so does “management”.

**Table 6:** Top 20 keywords in terms of its frequency for the “miRNAs in HD” research.

Rank	Keyword	Occurrences	Total link strength
1	Microrna	2,075	15,020
2	Expression	1,286	9,280
3	Heart failure	759	5,704
4	Myocardial infarction	664	5,064
5	Apoptosis	654	4,885
6	Biomarker	639	4,798
7	Heart	544	3,962
8	Circulating microrna	461	3,664
9	Disease	392	2,852
10	Cardiovascular disease	370	2,998
11	Cell	363	2,579
12	Mechanism	347	2,595
13	Proliferation	335	2,484
14	Gene expression	332	2,471
15	Hypertrophy	329	2,517
16	Activation	315	2,249
17	Cardiac hypertrophy	309	2,464
18	Cancer	278	2,021
19	Inflammation	268	1,902
20	Cardiomyocytes	261	1,971

**Table 7:** Top 20 keywords with the strongest citation bursts of the “miRNAs in HD” research.

Keywords	Year	Strength	Begin	End	2004–2023
Serum response factor	2005	8.35	2005	2012	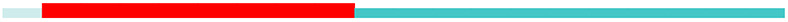
Muscle specific microrna	2007	26.68	2007	2014	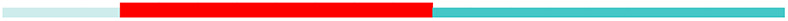
*In vivo*	2007	20.4	2007	2015	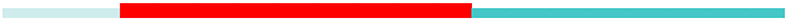
Dicer	2007	13.8	2007	2013	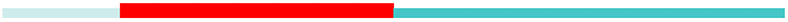
Signature	2007	10.69	2007	2015	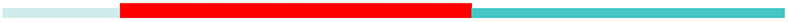
Human heart	2007	10.51	2007	2012	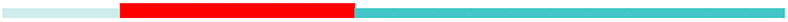
Mouse	2008	10.29	2008	2013	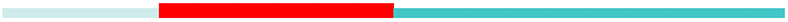
Vascular integrity	2008	9.7	2008	2012	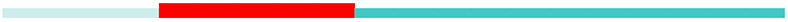
Messenger rna	2009	11.77	2009	2016	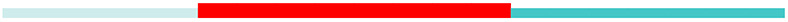
Mir 133	2009	8.74	2009	2015	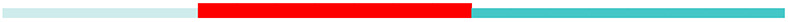
Gene expression	2005	15.15	2010	2011	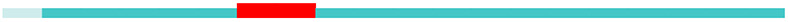
Targets	2006	8.72	2010	2014	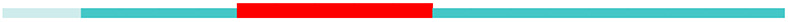
Autophagy	2018	11.76	2018	2023	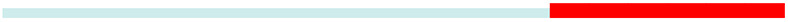
Long noncoding rna	2018	8.38	2018	2023	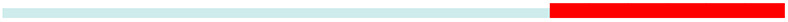
Diabetic cardiomyopathy	2018	12.68	2019	2023	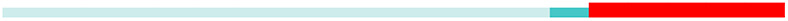
Long noncoding rnas	2019	12.51	2019	2021	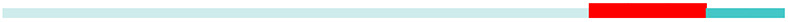
Cardiac dysfunction	2019	10.2	2019	2023	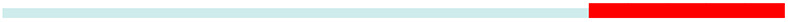
Extracellular vesicles	2016	9.77	2019	2023	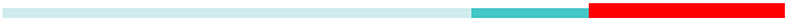
Exosomes	2015	10.66	2020	2023	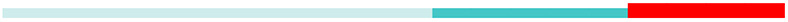

The value of citation burst strength (burst strength) reflects a significant increase in the citation frequency of a keyword within a specific time slice compared to other time slices, indicating that the keyword may become a research hotspot or trend during that period.

**Figure 8:**
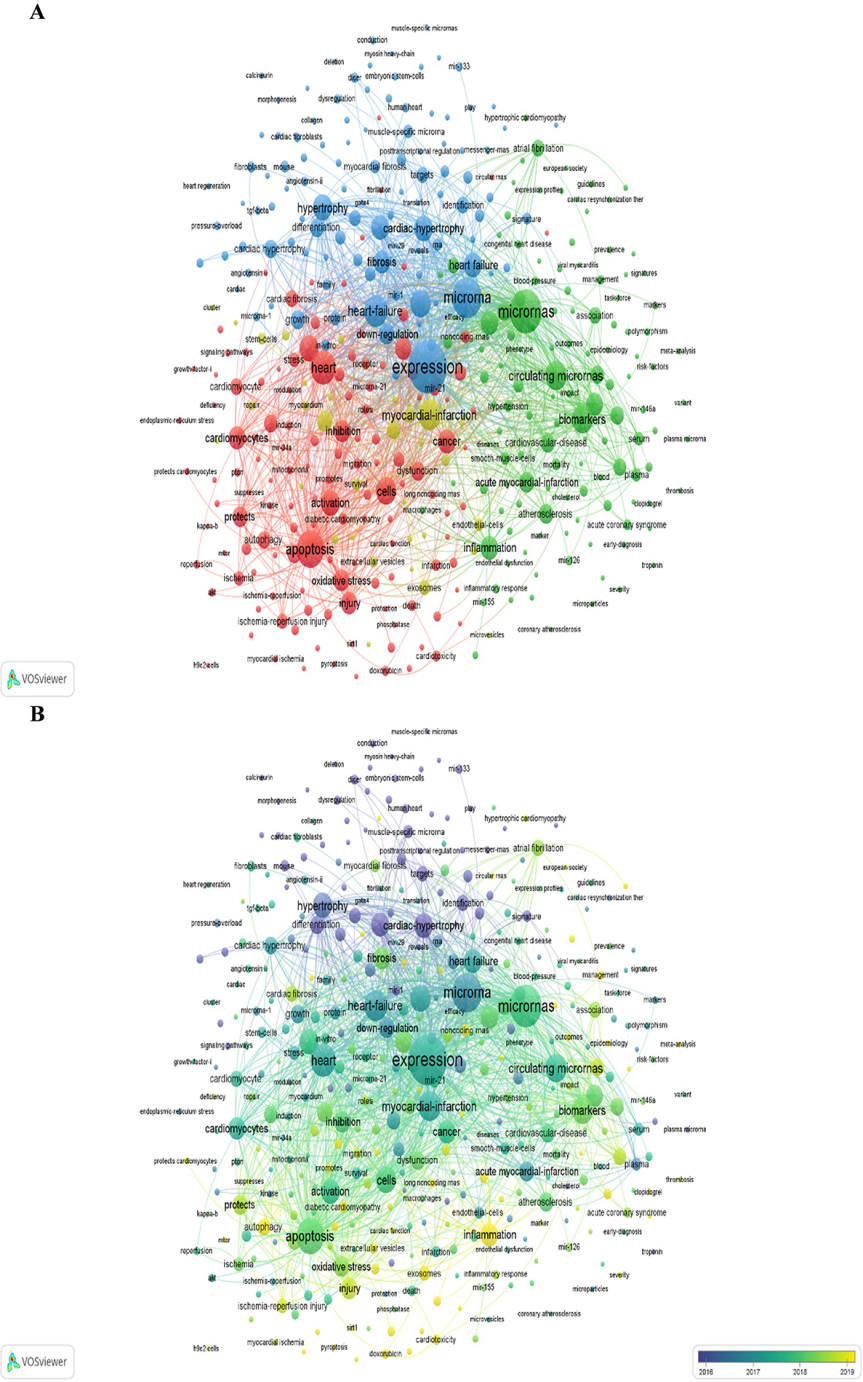
Keyword analysis of the "miRNAs in HD" research in VOSviewer. **(A)** Collaborative network visualization of keywords. **(B)** Keywords shift analysis over time. The color of a keyword node presents the average time of the keyword occurrence.

A co-occurrence network visualization of keywords with more than 15 occurrences is conducted in VOSviewer (Figure 8A). The lines between different keywords represent the co-occurrence relationship between them. The thickness of lines is proportional to the closeness of the connection between them. There are 5 clusters in terms of the study areas after excluding a single keyword as a cluster. One cluster links to the others with lots of lines, suggesting that they are intersecting areas in research directions. The blue cluster is related to potential therapy of HD (heart failure, diseases, expression, differentiation, fibrosis, down-regulation, hypertrophy, signature, identification, target, etc.). The yellow cluster is related to cell repair and angiogenesis (stem cells, endothelial cells, progenitor cells, angiogenesis, myocardial infarction, exosomes, repair, etc.). The green cluster is related to the relationship between cardiovascular and miRNAs (inflammation, cardiovascular diseases, circulating miRNAs, biomarker, impact, association, risk factors, etc.). The red cluster is related to cell death (apoptosis, autophagy, pyroptosis, dysfunction, etc.). Different clusters are linked with lots of lines, indicating that they are cross-cutting areas in each research direction.

Figure 8B marks the keywords with different colors according to the average year of their occurrences. The yellow keywords occurred later than the blue and purple ones. The color changes from purple to blue, then blue to green, and green to yellow indicating the dynamic development of the keyword over time. The size of the node indicates the keyword frequency, and the color of it implies the research focal points during a certain time. The result shows that “injury”, “inflammation”, “exosomes”, “cardiotoxicity”, “doxorubicin”, “sepsis”, “autophagy”, “pyroptosis”, etc., are novel research topics lately, in line with Table 7.

Overall, the “miRNAs in HD” research focused on the potential mechanisms, regulatory pathways, and expression change of specific miRNAs in various heart biological/pathological processes including autophagy, inflammation, and programmed death such as pyroptosis and apoptosis, especially in cancer, sepsis, injury situation. Consequently, in the twenty years, most of the research in this field is still in the basic stage of exploration, and the research hotspots are not highly concentrated. However, “exosomes”, “extracellular vesicles”, “autophagy”, and “management” have been novel hot research topics since 2018, which focused on the diagnosis and treatment of HD.

### 3.9 Highly cited publications analysis

Articles with high citation ranks have played a major role in the development of this research field. Hence, we analyzed the highly cited publications. The top 10 publications by citation frequency were ranked in Supplementary Table 1. The most frequently cited article was “Dysregulation of microRNAs after myocardial infarction reveals a role of miR-29 in cardiac fibrosis” (1,510) by Van Rooij, Eva, et al., which focused on the miR-29 family role as a regulator of cardiac fibrosis after myocardial infarction (12). The article titled “MicroRNA-133 controls cardiac hypertrophy” (1,464) by Care, Alessandra, et al, ranked second in the table, which focuses on the possible functional role of miRNAs in cardiac hypertrophy (13). The result showed that miR-133, and possibly miR-1 are key regulators of cardiac hypertrophy. The article titled “Control of stress-dependent cardiac growth and gene expression by a microRNA” (1,329) by Van Rooij, Eva, et al., focused on the miR-208 role in the cardiac stress response and ranked third (14). It is noting that another article by Van Rooij, Eva, et al., ranked fifth in the table, titled “A signature pattern of stress-responsive microRNAs that can evoke cardiac hypertrophy and heart failure” (1,251), focusing on the miR-195 role as a stress-inducible miRNA in cardiac hypertrophy (15).

The top four articles found that some miRNAs were potential targets for HD by measuring miRNA expression in both mice or rats and human hearts *in vivo*. Three of the top ten articles by citation frequency by the same author, Van Rooij, Eva, indicated the important role of the author in the field. The almost equally noteworthy writer in the table was Zhao Y, from the Gladstone Institute of Cardiovascular Disease, having two articles, ranking fourth and sixth, respectively. His articles entitled “Serum response factor regulates a muscle-specific microRNA that targets Hand2 during cardiogenesis” (2005), and “Dysregulation of cardiogenesis, cardiac conduction, and cell cycle in mice lacking miRNA-1-2” (2007), also introduced important basic research of miRNA regulation mechanism in this field (16, 17).

In addition, China, Italy, Germany, and the Netherlands have one article in the top 10 articles by citation frequency, respectively. Out of the ten references, six are published by the USA, indicating the pivotal role of the USA in miRNA-related basic research in HD again.

## 4 Discussion

The incidence and mortality rates for HD have remained high in the past decades, which is one of the global medical issues (18). Current evidence proves that specific miRNAs can modulate cardiac-embryo development and cardiac remolding in various ways (3, 19). miRNA-related research has gradually flourished since the first miRNA (lin-4) was discovered in C. elegans in 1993 (20, 21). The role of miRNAs continues to become definite with the research development, although many unknowns remain. As critical regulatory mediators of various biological and pathophysiological processes, miRNAs play an important role in cardiac development and diseases. It has been a trend to transform “miRNAs in HD” research achievement into clinical application in the current prognostication, diagnosis, and treatment of HD.

The “miRNA in HD” research has discovered some miRNAs that possess tissue and disease specificity, such as miR302 (22), miR-411 (23), and MiR-181a (24), etc. in myocardial infarction, miR-150-5p (25), miR-132 (26), miR-122 (27) and miRNA-129-5p (28), etc. in heart failure (HF), and miR-222 (29), miR-133 (13), and miR-155 (30), etc. in cardiac hypertrophy. Scientists described a new mode of regulating heart conditions, pointing to the potential diagnostic, prognostic, and therapeutic application of miRNAs for HD. Hence, miRNAs have been implicated the hope in the treatment of HD.

Some of the novel therapeutic approach of targeting specific miRNA, known as miRNA-targeted therapeutic, was proven successful in many preclinical studies for HD, with miRNA mimics carrying miRNA sequences or miRNA inhibitors (mainly antisense oligonucleotides, miRNA sponges, and erasers (3, 22, 24, 31–36). miRNA mimics and miRNA inhibitors were respectively designed to achieve therapeutic goals by upregulating or downregulating miRNA expression and then altering the target gene silencing effect. The challenges that miRNA-targeted therapeutic confront are the lack of safety, efficacy, stability, targeting specificity, delivery efficiency, etc. The innovation of the novel delivery systems, including chemical modification, the structure of binding them to small molecular substances (such as cholesterol and vitamin E), and appropriate delivery carriers (such as liposomes and nanocarriers) can only improve the above defects of miRNA mimics or inhibitors to different extents. Therefore, perfectly transforming miRNAs-target therapeutics into clinical applications is still difficult.

Some new techniques have recently overcome the stability and target-gene escape by suitable delivery systems and chemical modification. Gu et al. utilized exosomes loaded with miR302 through the cardiomyocyte-specific peptide to reduce myocardial ischemia and reperfusion (I/R) injury (22). Zhi et al. utilized the effective delivery of hypertrophic miR-182 inhibitor by cholesterol-containing nanocarriers to prevent pressure overload-induced cardiac hypertrophy (33).

To date, miRNA-related research has advanced into the clinical trial phase or clinical application for various diseases (37, 38), with a few for HD (26, 39). MiR-132 can cause myocardial remodeling through its high level of expression and its effect on signaling pathways in heart tissue, resulting in HF (26, 40). CDR132l, a miR-132 inhibitor, known as a milestone breakthrough in HF treatment, can attenuate or reverse HF (41). Its phase 1b clinical trial has confirmed that CDR132l is a relatively safe and well-tolerated miRNA therapeutic in stable chronic HF patients for the first time, without obvious toxicity (41). Although this study is limited by the small sample, the result of miRNA therapeutic effect in humans for HD is encouraging. Besides, the trial named HF-REVERT (Phase 2, multicenter, randomized, parallel, 3-arm, placebo-controlled Study to Assess Efficacy and Safety of CDR132l in Patients with Reduced Left Ventricular Ejection Fraction after Myocardial Infarction) is in progress, which aims to further evaluate the efficacy and safety of CDR132l in HF patients after acute myocardial infarction (42).

Interestingly, observational studies have identified specific geographical patterns in the exploration of miRNAs as diagnostic and prognostic biomarkers, as well as therapeutic agents, for cardiovascular diseases. These patterns suggest that differences in disease prevalence and genetic heterogeneity among regions may influence the direction and outcomes of research efforts.

The United States emphasizes the close integration of basic research and clinical applications, taking a leading position in the research and development of therapeutic targets and drugs for cardiovascular diseases that use miRNA. Researchers conduct in-depth studies on the mechanism of miRNA in heart development, often utilizing advanced gene-editing technologies and animal models (43, 44). They engage in a cross-disciplinary integration of fields such as molecular biology, genetics, and cardiology. These researchers not only achieve continuous breakthroughs in basic theoretical research but also actively collaborate with pharmaceutical companies to advance drug development.

Europe focuses more on exploring the role of miRNA in the pathogenesis of common heart diseases such as coronary heart disease and cardiomyopathy, as well as the application of miRNA as biomarkers in disease diagnosis and prognosis assessment (45). For example, through large-scale clinical sample analysis, researchers seek specific miRNA combinations as indicators for diagnosing coronary heart disease or evaluating the prognosis of cardiomyopathy (3). In the field of cardiac regeneration, studies investigate how miRNA regulates the proliferation and differentiation of cardiomyocytes, offering new insights into treating diseases like myocardial infarction. Some universities, with research teams that prioritize international cooperation, jointly undertake large-scale research projects. In their research, they excel at using systems biology methods to integrate multi-omics data and comprehensively analyze the role of miRNA in heart diseases (46, 47).

China centers on an extensive association between miRNA and cardiovascular diseases, covering various conditions such as arrhythmia, myocardial ischemia, cardiac hypertrophy, and heart failure (17, 48, 49). At the same time, China is also actively exploring the development of miRNA-based diagnostic methods and treatment strategies. For example, researchers in China are studying the value of miRNA in early disease diagnosis and investigating the use of traditional Chinese medicine and other methods to regulate miRNA expression for the treatment of cardiovascular diseases (50–52). The miRNA-related research has created a new era for Chinese traditional medicine in global clinical practice based on combining it with new scientific techniques.

Other Asian Countries like Japan and South Korea also have certain strengths in miRNA research. They keep up with the international frontiers in terms of technical applications and basic research (53–55). At the same time, they carry out relevant research in combination with the disease characteristics and research advantages of their own countries, such as studying the mechanism of miRNA in hypertensive heart disease (56, 57).

Australia has also achieved certain results in miRNA research on cardiovascular diseases. Its research teams pay attention to international cooperation and have carried out in-depth study on the epigenetic regulation of miRNA in heart diseases (58, 59).

## 5 Conclusion

Our study visualized the “miRNAs in HD” research in the past twenty years. We provided a comprehensive overview of this field based on different levels. The analyses of countries/regions, journals, and authors provided the research distribution, and the keyword analysis offered important clues about the research hots, frontiers, and trends.

China and the USA are in dominant positions in this field, with the maximum number of publications and total citations, respectively. However, the USA has established a leading role regarding the higher average citation and betweenness centrality than China. Peking Union Medical College is the most prolific university with the maximum publications, and University of California System is the most authoritative institution regarding betweenness centrality. PLOS ONE, Circulation Research, Journal of Molecular and Cellular Cardiology, and Scientific Reports, are the popular journals in this academic field. The International Journal of Molecular Sciences is the most popular journal for this academic field in 2023 with 26 articles. Thomas with 47 articles is the most prolific author. Van Rooij, Eva has the most frequently cited article, titled “Dysregulation of microRNAs after myocardial infarction reveals a role of miR-29 in cardiac fibrosis”, which suggests his important influence in this field. Considering the number of publications, citations, and total link strength overall, Olson. Eric N, Van Rooij Eva, Thum Thomas; Yang Baofeng, Wang Kun; and Lu Yanjie are authoritative authors in this field.

The study shows that the researchers in this field have been continuously devoted to basic research, and the research hotspots have not been highly concentrated during the past twenty years. The expression changes and regulatory mechanisms of specific miRNAs in various heart biological and pathophysiological processes have always been this field's research direction. The development trend in this field is how to transform the current achievements into clinical applications. Although the researchers in this field have obtained preliminary success, there is a long way to clinical practice.

The investigation into miRNAs has introduced a groundbreaking approach to the regulation of gene expression following transcription. Moreover, circulating miRNAs hold immense promise as diagnostic and prognostic biomarkers. However, to confirm their dependability, a multitude of challenges must be tackled. The challenges encompassing accurate detection and analysis techniques, the standardization of biological samples, the validation of miRNA function, the transition to clinical applications, the sharing and integration of data, accounting for individual variations, and the appropriate selection of experimental models all demand urgent attention and resolution.

The promise of miRNA-based therapeutic approaches is indeed captivating, yet significant challenges remain to be conquered. Delving into the target genes of microRNAs and understanding their functions is pivotal to crafting miRNA-based interventions for a range of human diseases. The creation of miRNA mimetics and inhibitors stands out as a promising strategy for either restoring function or counteracting endogenous miRNAs. However, the administration of high doses of these synthetic miRNA analogs can inadvertently trigger an innate immune response, leading to an unwarranted surge in cytokine expression. Furthermore, extensive research is ongoing to enhance the stability of miRNA mimics and inhibitors during the delivery process. Addressing these issues is essential for the successful future implementation of miRNA-based treatments.

Multidisciplinary team collaboration, interdisciplinary studies and emerging technologies may provide hope for further development of miRNA research in heart diseases. The application of emerging technologies such as nanotechnology, proteomics, and metabolomics, artificial intelligence, and machine learning can help promote research on miRNA in the field of heart diseases, enhance our understanding of heart diseases, and provide possibilities for the development of new diagnostic and therapeutic strategies. For example, nanotechnology (including liposomes, nanocarriers, and exosomes) addresses the challenge of delivering miRNA mimics and inhibitors, which have help transform miRNA research into clinical application. In this article, researchers have the chance to identify potential collaborators and key hotspots that align with their interests, which is in line with my writing intention.

## 6 Limitations

By using bibliometric analysis, our study provides better insight into research frontiers and focus, but several inherent limitations are unavoidable. First, the raw data downloaded only from the WOSCC database has selective bias. However, compared to other data resources, the WOSCC database can provide more comprehensive and adequate information (9). Second, our study only included English articles, and we may have deleted some important articles published in other languages.

However, our study has incorporated the majority of “miRNAs in HD” research from 2004 to 2023, and we believe that the results would not be different even inclusion of new articles. The interplay between miRNA and various HD is still under intense investigation. There are many questions not well understood and require further investigation. In addition, research on “miRNAs in HD” may exhibit heterogeneity, necessitating further studies to replicate the research findings in order to identify reliable results. The potential of miRNA as a diagnostic tool, prognostic indicator, and therapeutic target for HD still requires validation in independent, large-scale cohorts.

## Data Availability

The datasets presented in this study can be found in online repositories. The names of the repository/repositories and accession number(s) can be found in the article/Supplementary Material.
