# Supplementary material for: Bibliometric and visual analysis of miRNAs in heart diseases from 2004 to 2023
Source: Front Cardiovasc Med. 2025 Mar 20;12:1465646. doi: 10.3389/fcvm.2025.1465646 (PMC11965657; doi:10.3389/fcvm.2025.1465646)
Supplement: Supplementary file 1 [file Table1.pdf]

SUPPLEMENTARY TABLE 1 Top 10 highly cited publications of miRNAs-related research in HD.

Rank	Article Title	Source	First Author	Year	Cited	DOI
1	Dysregulation of microRNAs after myocardial infarction reveals a role of miR-29 in cardiac fibrosis	Biological Sciences	Van Rooij, Eva	2008	1510	10.1073/pnas.0805038105
2	MicroRNA-133 controls cardiac hypertrophy	Nature Medicine	Care, Alessandra	2007	1464	10.1038/nm1582
3	Control of stress-dependent cardiac growth and gene expression by a microrna	Science	Van Rooij, Eva	2007	1329	10.1126/science.1139089
4	Serum response factor regulates a muscle-specific microRNA that targets Hand2 during cardiogenesis	Nature	Zhao,Y	2005	1309	10.1038/nature03817
5	A signature pattern of stress-responsive microRNAs that can evoke cardiac hypertrophy and heart failure	Proceedings of The National Academy of Sciences of The United States of America	Van Rooij, Eva	2006	1251	10.1073/pnas.0608791103
6	Dysregulation of cardiogenesis, cardiac conduction, and cell cycle in mice lacking miRNA-1-2	Cell	Zhao, Y	2007	1112	10.1016/j.cell.2007.03.030
7	Circulating micrornas in patients with coronary artery disease	Circulation Research	Fichtlscherer, Stephan	2010	1003	10.1161/CIRCRESAHA.109.215566
8	Pervasive roles of microRNAs in cardiovascular biology	Nature	Small, Eric M	2011	969	10.1038/nature09783
9	The muscle-specific microRNA miR-1 regulates cardiac arrhythmogenic potential by targeting GJA1 and KCNJ2	Nature Medicine	Yang, Baofeng	2007	915	10.1038/nm1569
10	Circulating microRNAs novel biomarkers and extracellular communicators in cardiovascular disease?	Circulation Research	Creemers, Esther E	2012	812	10.1161/CIRCRESAHA.111.247452
